# Inclusion of clavulanic acid determinants in the basophil activation test improves the evaluation of immediate reactions to amoxicillin-clavulanic acid

**DOI:** 10.1186/2045-7022-4-S3-P32

**Published:** 2014-07-18

**Authors:** Tahia D Fernandez, Adriana Ariza, Maria Salas, Maria I Montañez, Inmaculada Doña, Lidia Melendez, Maria D Cañamero, Miguel Blanca, Maria J Torres, Cristobalina Mayorga

**Affiliations:** 1 IBIMA-Regional University Malaga Hospital-UMA, Research Laboratory, Spain; 2 IBIMA-Regional University Malaga Hospital-UMA, Allergy Service, Spain; 3 Regional University Malaga Hospital, Allergy Service, Spain

## Background

The clavulanic acid (CLV) is nowadays frequently included in the treatment with Amoxicillin (AX). This component of the antibiotic therapy initially thought to have a low immunogenic capacity; however immediate allergic reactions to CLV have been reported in a 30% of patients allergic to AX-CLV. Basophil activation test (BAT) has shown promising results demonstrating specific recognition of CLV determinants. The aim of this study was to assess the value of BAT in the evaluation of immediate allergic reactions to CLV.

## Method

Patients with a strong clinical history of having suffered an immediate reaction after AX-CLV administration were evaluated. The allergological study followed the European Academy guidelines, included skin test with penicillin G, AX, and CLV determinants and drug provocation test when indicated. BAT was carried out using AX and CLV at different concentrations (2.5, 1.25, 0.25 and 0.05 mg/ml).

## Results

Among 75 patients included, 64 were finally diagnosed as allergic, 26 to AX and 38 to CLV. The sensitivity of BAT was 60% and the specificity 81.8%. The inclusion of AX determinant produced a BAT sensitivity of 54.1% whereas CLV determinant produced a BAT sensitivity of 78.6% with a specificity of 91% and 82% respectively. In patients diagnosed as allergic to AX the BAT sensitivity was 50% whereas in patients allergic to CLV, BAT sensitivity was 65.8%.

## Conclusion

The inclusion of clavulanic acid in the basophil activation test increases its diagnostic capacity in patients with immediate allergic reaction to the combination amoxicillin-clavulanic acid.

